# Chlaralose

**Published:** 1894-01-27

**Authors:** 


### NEW DRUGS AND PREPARATIONS.

[We shall be glad to receive, at our Office, 428, Strand, London, W.C., from the manufacturers, specimens of all new preparations and drugs which may be brought out from time to time.]

### CHLORALOSE.

This new hypnotic was recently introduced by Professor Richet and Dr. Hanriot, of Paris. It is a combination of chloral and glucose. After taking precautions to ascertain its non-poisonous properties in small doses, the discoverers took it in doses increasing from ten to seventy-five centigrammes, and found that while it was an excellent hypnotic in doses of ten centigrammes, even the very large amounts they themselves took were not harmful. They consider that in addition to its utility as a safe hypnotic, its specific excitant action on the medulla and spinal cord will render it a valuable remedy in cases of neurasthenic, neuralgic, and other painful affections, and in tabes and perhaps in diabetes. Richet and Henriot believe that the drug acts on the grey matter of the cerebral hemispheres, diminishing their activities. It has a hypnotic action on the cerebrum, but is an excitant of the medulla. A case of poisoning by chloralose recently reported by Dr. Lang would serve to show that it does not give rise to any definite psychic phenomena beyond loss of consciousness.

The patient, he was told, had been in the habit of taking hypnotics. On his arrival he found a middle-aged woman, lying in bed in a semi-comatose condition. She showed signs of irritation on attempts being made to wake her, but she could not be roused sufficiently to give an intelligible answer to any question. The face was congested and bluish, pupils equal and somewhat dilated, breathing normal, pulse 60. Fearing the amount taken might have been considerable, he washed out the stomach and gave an enema of hot coffee. She had taken six and a half grains of chloralose at one a.m., and three and a half at two a.m. Dr. Lang saw her at half-past four a.m. She soon recovered, with the exception of a slight headache. This case will act as a caution to those who have accepted the statement that chloralose is a "perfectly" safe hypnotic.

*Pharmacology.*—Its bitter taste is somewhat difficult to overcome, thus even were the drug readily soluble in water, it would be an unpleasant remedy dissolved in the form of a draught. It should be given in cachets or capsules. It may be given as a powder suspended in milk. The minimum dose for adults is ten centigrammes, but this is a weak dose, and generally there is no harm in increasing it to forty or even sixty centigrammes.

The drug has been used in Professor Grasset's clinic, and Dr. Sacaze has summarised his results[2].

Chloralose was administered as a hypnotic in cases of neurasthenia, locomotor ataxy, diabetes, pulmonary tuberculosis, pseudo-tabes, due to neuritis, and cerebral tumour. It produced sleep in every case, but in different doses. The maximum of ten grains was, however, never exceeded. It was subsequently observed that several of the patients continued to sleep well although the dose was diminished, or even after the administration was entirely stopped. It would appear, therefore, that under the influence of this substance sleep is not only induced for a time, but that the habit, as it were, becomes restored. The patients usually complained of no after effects.

In two or three of these cases a remarkable improvement was observed in the night sweats. No change was observed in the urine of the diabetic case. There was no digestive disturbance in any case for the administration of chloralose. The remedy was given in cachets, but when given in capsules the patients became restless and drowsy and suffered from nightmare. Dr. Sacaze explains the difference by the supposition that when given in capsules the drug passes into the circulation in small quantities at a time, when it acts as a stimulant, whereas when taken in cachets, absorption takes place rapidly.

In a case of tetanus the first dose of chloralose produced a marked effect in diminishing the spasm, deglutition became easier, and cramp disappeared. The patient became drowsy, and sleep returned on the second day. The dose administered varied from twelve to eighteen grains. He died after five days from hyperpyrexia.

Basset, reporting the results of his observations, found it produced light, refreshing sleep, and that on awaking there is none of that heaviness which persists for some time after the preparations of chloral, nor is there constipation and loss of appetite as after opium in its various forms. He cautions practitioners of the necessity for care in giving it in organic disease, and considers it is even contra-indicated in tabes.

Further, Maragliano[4] has presented a report of his experience of chloralose. He found it was an eminently satisfactory hypnotic. Sleep generally was obtained within half an hour of its administration, and was quiet and without dreams and nightmare. During sleep sensitiveness to pain was lessened, sometimes to complete anæsthesia, sometimes only diminished, the result depending on the amount of the dose employed, other things being equal. Reflexes were exaggerated. Thus those patients who were subject to continuous cough were liable to have the symptom exaggerated. It gave rise to no unpleasant after effects, nor was the pulse, respiration, or the digestive tract affected. In about half his cases there was a slight temporary headache on awaking. Sometimes, however, he found it gave rise to muscular tremor and to a sort of cataleptic condition.

At a recent meeting of the Academy of Medicine of Turin, MM. Murro and Lombrozo[5] reported results obtained from chloralose in ten cases of three lunatics. The insomnia ceased on the first night the drug was given. But while 0·25 grammes was a sufficient dose the first night, it had to be increased to 0·50 on the following evening, in order to get the effect. On the discontinuance of the drug the patients fell back to their previous condition, and the sleeplessness reappeared. It produced a lowering of temperature. They also studied the effect of the drug on the urine, and found that urea was generally diminished, and the chlorides always decreased. The diminution in chlorides they considered was especially interesting as indicating that there was a close analogy between the artificial sleep produced by chloralose and physiological sleep.

Lombrozo further adds that he considers chloralose the least harmful of all hypnotics, and free from all the risks of chloral. It very seldom produces constitutional disturbances, but Lombrozo observed tremor and loss of memory, itching, in one case after doses of 0·15 to 0·20 grammes.

D'Amore[6] too has reported well of the drug after an experience of fifteen cases. He found that in doses of four and a half to fifteen grains it gave prolonged and restful sleep. In some of the cases chloral had failed to overcome the insomnia.

The results obtained and reported by MM. Landouzy, Martin, Marie, and Légard are so similar to the observations already reviewed, that further repetition is unnecessary.

[1] Les Nouv. Rém., Jan. 24, 1893. [2] La Lem. Méd, April 7, 1893. [3] Union Méd., Sept. 2, 1893. [4] Les Nouv. Rém., June 24, 1893. [5] Gazz. d. Osp., June 10, 1893. Les Nouv. Rém., Aug. 8, 1893. [6] Gazz. d. Osp., 1893, No. 65.

